# Supplementary material for: Unravelling the Molecular Determinants of Bee Sensitivity to Neonicotinoid Insecticides
Source: Curr Biol. 2018 Apr 2;28(7):1137–1143.e5. doi: 10.1016/j.cub.2018.02.045 (PMC5887109; doi:10.1016/j.cub.2018.02.045)
Supplement: Document S1. Figures S1–S3 and Tables S1–S3 [file mmc1.pdf]

Current Biology, Volume *28*

Supplemental Information

Unravelling the Molecular Determinants

of Bee Sensitivity to Neonicotinoid Insecticides

Cristina Manjon, Bartlomiej J. Troczka, Marion Zaworra, Katherine Beadle, Emma Randall, Gillian Hertlein, Kumar Saurabh Singh, Christoph T. Zimmer, Rafael A. Homem, Bettina Lueke, Rebecca Reid, Laura Kor, Maxie Kohler, Jürgen Benting, Martin S. Williamson, T.G. Emyr Davies, Linda M. Field, Chris Bass, and Ralf Nauen

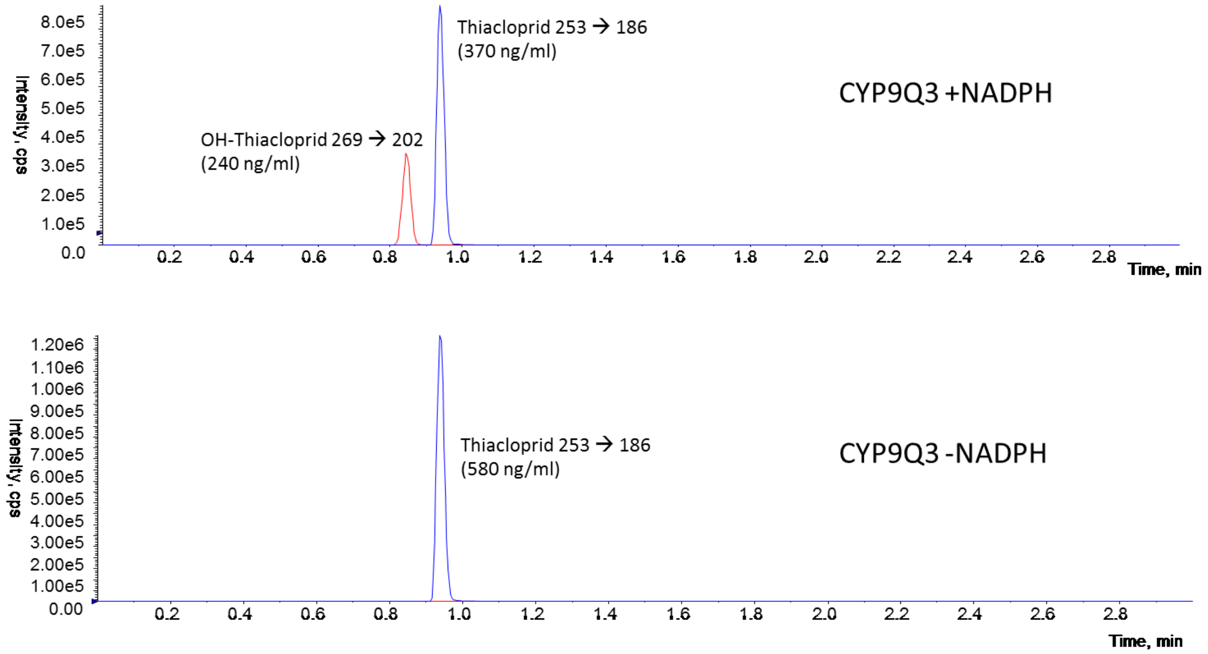

Figure S1. LC-MS/MS analysis of thiacloprid metabolism by CYP9Q3. Related to Figure 2. LC-MS analysis of thiacloprid metabolism. Typical MRM chromatograms of the CYP9Q3 catalysed formation of OH-thiacloprid with and without NADPH. Ion transition of thiacloprid $[M+H]^+$ 253 and OH-thiacloprid $[M+H]^+$ 269 to their fragments m/z 186 and m/z 202 are measured, respectively.

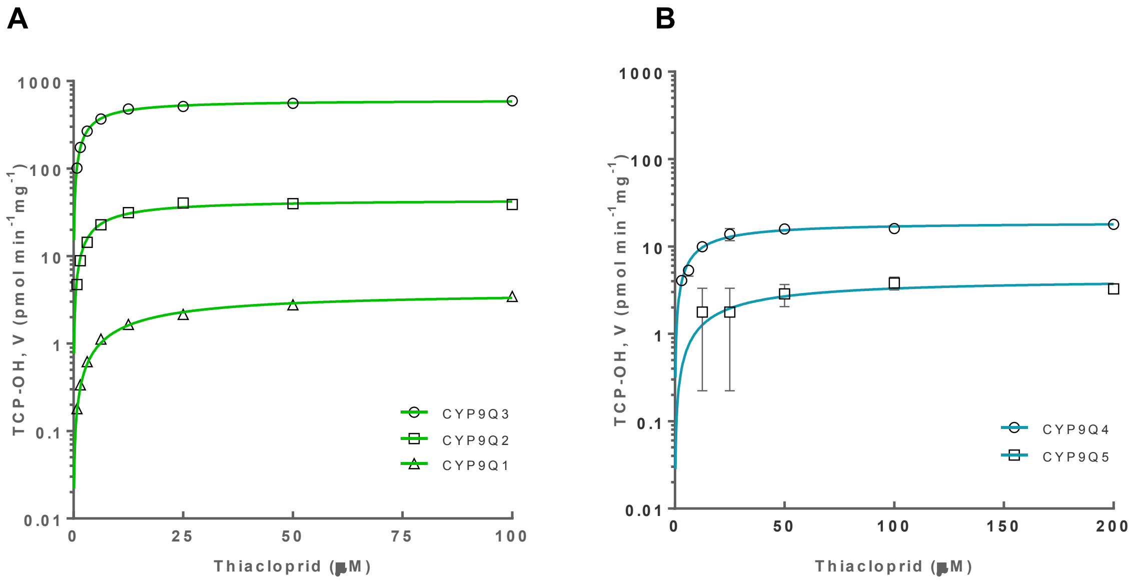

	CYP9Q1	CYP9Q2	CYP9Q3
V_{max}	3,92 ±0,08	44,31±1,01	607,8±5,48
K_m	17,83±1,08	5,65±0,49	3,92±0,14

	CYP9Q4	CYP9Q5
V_{max}	18,94±0,64	4,29±0,71
K_m	11,82±1,51	30,04±14,85

Figure S2. Michaelis-Menten kinetics of thiacloprid hydroxylation by *A. mellifera* and *B. terrestris* metabolising P450s analysed by non-linear regression. Related to Figure 2. A, B, Michaelis-Menten kinetics plots of thiacloprid hydroxylation catalyzed by AmCYP9Q1-3 **(A)** and BtCYP9Q4-5 **(B)**. The apparent K_m and V_{max} values for thiacloprid are indicated below the respective graphs. Data points are mean values ± SD (n=3).

	Bt CYP9P1	Bt CYP9P2	Bt CYP9R1	Bt CYP9Q4	Bt CYP9Q5	Am CYP9Q1	Am CYP9Q3	Am CYP9Q2
Bt CYP9P1		63.953%	41.454%	37.795%	37.795%	37.305%	36.471%	38.235%
Bt CYP9P2	63.953%		41.765%	40.354%	40.748%	37.305%	37.451%	39.412%
Bt CYP9R1	41.454%	41.765%		42.185%	42.938%	38.716%	41.892%	42.402%
Bt CYP9Q4	37.795%	40.354%	42.185%		93.143%	55.340%	56.840%	61.163%
Bt CYP9Q5	37.795%	40.748%	42.938%	93.143%		54.563%	57.225%	61.726%
Am CYP9Q1	37.305%	37.305%	38.716%	55.340%	54.563%		53.488%	56.589%
Am CYP9Q3	36.471%	37.451%	41.892%	56.840%	57.225%	53.488%		57.500%
Am CYP9Q2	38.235%	39.412%	42.402%	61.163%	61.726%	56.589%	57.500%	

Figure S3. Heat map showing the levels of sequence identity between *A. mellifera CYP9Q1-3* and *B. terrestris CYP9* genes. Related to Figure 2.

Application	Insecticide	Synergist	LD_{50} (µg/bee)	95% CI	Slope	± SE	Synergism Ratio
**Topical**	Imidacloprid	None	0.38	0.12 - 1.45	0.6	0.11	n/a
	Thiacloprid	None	>100	n/a	n/a	n/a	n/a
**Oral**	Imidacloprid	None	0.038	0.012 - 0.075	1.5	0.44	n/a
		PBO	0.032	0.016 - 0.05	1.9	0.41	1.2
	Thiacloprid	None	19.68	13.45 - 26.88	1.8	0.26	n/a
		PBO	4.73	2.55 - 7.71	1.4	0.24	4.2

Table S1. Sensitivity of *Bombus terrestris* to imidacloprid and thiacloprid in insecticide bioassays. Related to Figure 1. Neonicotinoid acute contact and acute oral LD_{50} values (±95% confidence intervals) and slope (±SE) for *Bombus terrestris* 48 hours after application of insecticide. Synergism ratio is also shown, where the P450 inhibitor piperonyl butoxide (PBO) was used.

Primers	**Sequence**	**Use**
**AmCyp9Q3 F1**	5'-GATGTGCGTCGAGAGTTTCC-3'	qPCR (CYP9Q3)
**AmCyp9Q3 R1**	5'-CTGTCCGGGTCGAATTTGTC-3'	qPCR (CYP9Q3)
**AmCyp9Q2 F1**	5'-ATGGAAGGAGCACAGGAACA-3'	qPCR (CYP9Q2)
**AmCyp9Q2 R1**	5'-ACGTCGTTGGTGTATCTGGT-3'	qPCR (CYP9Q2)
**AmCyp9Q1 F1**	5'-GGAGGAGGGGAAGAGAGGTA -3'	qPCR (CYP9Q1)
**AmCyp9Q1 R1**	5'-CCTCCTGAAGCCTCTGTTGA-3'	qPCR (CYP9Q1)
**AmRpl32 F1**	5'-AGTAAATTAAAGAGAAACTGGCGTAA-3'	qPCR (reference gene)
**AmRpl32 R1**	5'-TAAAACTTCCAGTTCCTTGACATTAT-3'	qPCR (reference gene)
**AmGADPH F1**	5'-ACCTTCTGCAAAATTATGGCGA-3'	qPCR (reference gene)
**AmGADPH R1**	5'-CACCTTTGCCAAGTCTAACTGTTAAG-3'	qPCR (reference gene)
**BtCyp9Q4 F1**	5'-TATTCCACCAACGCCACTGT-3'	qPCR (CYP9Q4)
**BtCyp9Q4 R1**	5'-GGTCCACTTCCTTGTATGCG-3'	qPCR (CYP9Q4)
**BtCyp9Q5 F1**	5'-CCTACGATGCTCTAAGCGAGATG-3'	qPCR (CYP9Q5)
**BtCyp9Q5 R1**	5'-ATTCTCGTAATATTGAGGATCGCG-3'	qPCR (CYP9Q5)
**BtPal F1**	5'-TGTCGGTATCTACGCGCCTG-3'	qPCR (reference gene)
**BtPal R1**	5'-TTGGTGGATGCTTGTCAGTC-3'	qPCR (reference gene)
**BtEEF1A F1**	5'-AGAATGGACAAACCCGTGAG-3'	qPCR (reference gene)
**BtEEF1A R1**	5'-CACAAATGCTACCGCAACAG-3'	qPCR (reference gene)
**D099 pUAST F**	TCACTGGAACTAGGCTAGCA-3'	Sequence validation of transgenic flies
**D102 pUAST F**	5'-GGATCCAAGCTTGCATGCCTG-3'	sequence validation of transgenic flies
**D100 pUAST R**	5'-AAAGGCATTCCACCACTGCT-3'	sequence validation of transgenic flies
**D101 pUAST R**	5'-CCACCACTGCTCCCATTCAT-3'	sequence validation of transgenic flies
**AmCyp9Q3 F3**	5'-TGGAAGGAGCACAGGAACAT-3'	in situ hybridisation (CYP9Q3)
**AmCyp9Q3 R6-T7**	5'-TAATACGACTCACTATAGGGAGATGATCACGGCGTCCATGTAT-3'	In situ hybridisation (CYP9Q3)

Table S2. Sequence of oligonucleotide primers for, PCR, qRT-PCR and *in situ* hybridization used in this study. Related to STAR methods.

Species	Gene name	Accession Number
Bombus terrestris	CYP9Q4	XP_003393377
Bombus terrestris	CYP9Q5	XP_003393376.1
Bombus terrestris	CYP9P1	XP_020718545.1
Bombus terrestris	CYP9P2	XP_003393388.3
Bombus terrestris	CYP9R1	XP_003393379.1
Apis mellifera	CYP9Q1	XP_006562364
Apis mellifera	CYP9Q2	XP_392000
Apis mellifera	CYP9Q3	XP_006562363
Apis mellifera	CYP9R1	GB16803
Apis mellifera	CYP9S1	XP_016771487
Apis mellifera	CYP336A1	XP_001119981
Apis mellifera	CYP9P1	XP_006562365
Apis mellifera	CYP9P2	GB19055
Apis mellifera	CYP6AQ1	NP_001191991
Apis mellifera	CYP6AR1	XP_623362
Apis mellifera	CYP6AS1	GB16899
Apis mellifera	CYP6AS2	GB19197
Apis mellifera	CYP6AS3	GB15681
Apis mellifera	CYP6AS4	XP_395671
Apis mellifera	CYP6AS5	DQ232888
Apis mellifera	CYP6AS7	XP_006565064
Apis mellifera	CYP6AS8	XP_006565076
Apis mellifera	CYP6AS10	XP_016771320
Apis mellifera	CYP6AS11	XP_016771191
Apis mellifera	CYP6AS12	XP_397347
Apis mellifera	CYP6AS13	GB17831
Apis mellifera	CYP6AS15	XP_623595
Apis mellifera	CYP6AS17	XP_006565063
Apis mellifera	CYP6AS18	XP_006565063
Apis mellifera	CYP6BC1	XP_016766476
Apis mellifera	CYP6BD1	XP_006564499
Apis mellifera	CYP6BE1	XP_624795

Table S3. Accession numbers of P450 sequences functionally expressed in this study. Related to STAR methods.
