# Supplementary material for: Unravelling the Molecular Determinants of Bee Sensitivity to Neonicotinoid Insecticides
Source: Curr Biol. 2018 Apr 2;28(7):1137–1143.e5. doi: 10.1016/j.cub.2018.02.045 (PMC5887109; doi:10.1016/j.cub.2018.02.045)
Supplement: Document S2. Article plus Supplemental Information [file mmc2.pdf]

Current Biology

Report

Unravelling the Molecular Determinants of Bee Sensitivity to Neonicotinoid Insecticides

Highlights

- Honeybees and bumble bees show variation in sensitivity to different neonicotinoids
- Variation in bee sensitivity to neonicotinoids does not reside at the target site
- Cytochrome P450s of the CYP9Q subfamily determine bee sensitivity to neonicotinoids
- CYP9Q genes are highly expressed in bee Malpighian tubules and the brain

Authors

Cristina Manjon, Bartlomiej J. Troczka, Marion Zaworra, ..., Linda M. Field, Chris Bass, Ralf Nauen

Correspondence

c.bass@exeter.ac.uk (C.B.),
ralf.nauen@bayer.com (R.N.)

In Brief

N-cyanoamidine neonicotinoid insecticides are much less toxic to honeybees and bumble bees than *N*-nitroguanidine compounds. Manjon et al. show that this results from differences in their efficiency of metabolism by cytochrome P450s of the CYP9Q subfamily, demonstrating their role as key determinants of bee sensitivity to this insecticide class.

Manjon et al., 2018, Current Biology *28*, 1137–1143
April 2, 2018 © 2018 The Author(s). Published by Elsevier Ltd.
https://doi.org/10.1016/j.cub.2018.02.045

Current Biology
Report

CellPress

Unravelling the Molecular Determinants of Bee Sensitivity to Neonicotinoid Insecticides

Cristina Manjon,[1,5] Bartlomiej J. Troczka,[2,5] Marion Zaworra,[1,4,5] Katherine Beadle,[3] Emma Randall,[3] Gillian Hertlein,[1] Kumar Saurabh Singh,[3] Christoph T. Zimmer,[3,6] Rafael A. Homem,[2] Bettina Lueke,[1] Rebecca Reid,[2] Laura Kor,[2] Maxie Kohler,[1] Jürgen Benting,[1] Martin S. Williamson,[2] T.G. Emyr Davies,[2] Linda M. Field,[2] Chris Bass,[3,7,*] and Ralf Nauen[1,*]

[1]Bayer AG, Crop Science Division, Alfred Nobel-Strasse 50, 40789 Monheim, Germany
[2]Department of Biointeractions and Crop Protection, Rothamsted Research, Harpenden, UK
[3]College of Life and Environmental Sciences, Biosciences, University of Exeter, Penryn Campus, Penryn, Cornwall, UK
[4]Institute of Crop Science and Resource Conservation, Rheinische Friedrich-Wilhelms University Bonn, 53115 Bonn, Germany
[5]These authors contributed equally
[6]Present address: Syngenta Crop Protection, Werk Stein, Schaffhauserstrasse, Stein CH4332, Switzerland
[7]Lead Contact
*Correspondence: c.bass@exeter.ac.uk (C.B.), ralf.nauen@bayer.com (R.N.)
https://doi.org/10.1016/j.cub.2018.02.045

SUMMARY

The impact of neonicotinoid insecticides on the health of bee pollinators is a topic of intensive research and considerable current debate [1]. As insecticides, certain neonicotinoids, i.e., *N*-nitroguanidine compounds such as imidacloprid and thiamethoxam, are as intrinsically toxic to bees as to the insect pests they target. However, this is not the case for all neonicotinoids, with honeybees orders of magnitude less sensitive to *N*-cyanoamidine compounds such as thiacloprid [2]. Although previous work has suggested that this is due to rapid metabolism of these compounds [2–5], the specific gene(s) or enzyme(s) involved remain unknown. Here, we show that the sensitivity of the two most economically important bee species to neonicotinoids is determined by cytochrome P450s of the CYP9Q subfamily. Radioligand binding and inhibitor assays showed that variation in honeybee sensitivity to *N*-nitroguanidine and *N*-cyanoamidine neonicotinoids does not reside in differences in their affinity for the receptor but rather in divergent metabolism by P450s. Functional expression of the entire CYP3 clade of P450s from honeybees identified a single P450, CYP9Q3, that metabolizes thiacloprid with high efficiency but has little activity against imidacloprid. We demonstrate that bumble bees also exhibit profound differences in their sensitivity to different neonicotinoids, and we identify CYP9Q4 as a functional ortholog of honeybee CYP9Q3 and a key metabolic determinant of neonicotinoid sensitivity in this species. Our results demonstrate that bee pollinators are equipped with biochemical defense systems that define their sensitivity to insecticides and this knowledge can be leveraged to safeguard bee health.

RESULTS AND DISCUSSION

Bees carry out vital ecosystem services by pollinating wild plants and economically important crops but, in doing so, are exposed to a wide variety of natural and synthetic xenobiotics (including pesticides) [6]. Understanding the molecular defense systems that bees use to protect themselves from these potential toxins and their effectiveness and specificity provides important knowledge that can be used to avoid negative off-target effects [7].

Previous studies have demonstrated that honeybees exhibit marked differences in their sensitivity to different pesticides. Indeed, certain compounds display such low acute toxicity to bees that they are used as in-hive treatments by beekeepers against parasitic *Varroa* mites [6]. This differential sensitivity extends to neonicotinoid insecticides, with honeybees exhibiting profound differences in their sensitivity to N-nitroguanidine and N-cyanoamidine neonicotinoids [2]. In this study, we used imidacloprid and thiacloprid as exemplars of each class and first examined whether this differential sensitivity extends to bumble bees (*Bombus terrestris*), the second-most economically important bee pollinator species worldwide. In both contact and oral bioassays, significant (> 500-fold) differences were observed in the sensitivity of bumble bees to the two compounds (Figure 1A, Table S1). Based on these results and previous data for honeybees [9, 10], imidacloprid is categorized as "highly toxic" to both bumble bees and honeybees, according to the U.S. Environmental Protection Agency (EPA) (Figure 1A) [8]. In contrast, thiacloprid is categorized as "slightly toxic" or "practically non-toxic" to both bee species depending on the route of exposure (Figure 1A) [10].

The molecular basis of the differences in sensitivity of bees to these neonicotinoids could reside in differences in their affinity for the target site, the nicotinic acetylcholine receptor (nAChR), or from differences in the speed and efficiency of their metabolism. To examine the role of the former in intrinsic bee tolerance to thiacloprid, we carried out radioligand binding assays using honeybee and bumble bee head membrane preparations, an enriched source of nAChRs, using tritiated imidacloprid and examined the displacement of [^{3}H]-imidacloprid by both

 © 2018 The Author(s). Published by Elsevier Ltd.
This is an open access article under the CC BY license (http://creativecommons.org/licenses/by/4.0/).

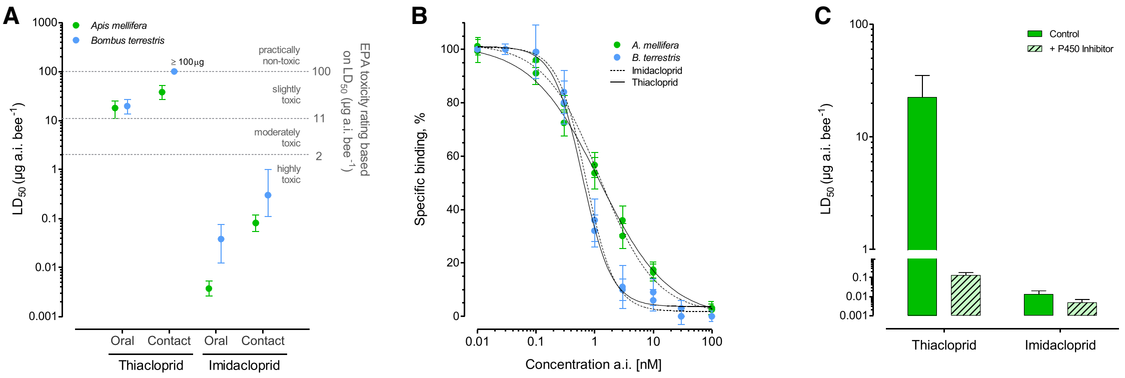

Figure 1. Toxicodynamics and Pharmacokinetics of Neonicotinoid Sensitivity in Two Bee Species

(A) LD_{50} values for imidacloprid and thiacloprid upon oral and topical application in *A. mellifera* and *B. terrestris*. Sensitivity thresholds are depicted according to EPA toxicity ratings [8]. Data for *A. mellifera* is taken from [9, 10], data for *B. terrestris* was generated in this study. Error bars display 95% CLs (n = 4).

(B) Specific binding of thiacloprid and imidacloprid to both *A. mellifera* and *B. terrestris* nAChRs. Error bars display standard deviation (n = 3).

(C) Sensitivity of *A-p-methoxy-. mellifera* to imidacloprid and thiacloprid before and after pretreatment with the insecticide synergist ABT (aminobenzotriazole). Error bars display 95% CLs (n = 3).

See also Table S1.

unlabelled imidacloprid and thiacloprid. As shown in Figure 1B, in the case of both bee species, both imidacloprid and thiacloprid bind with nM affinity, and no significant difference was seen in the specific binding of either compound at the receptor (IC_{50} of 1.2, [95% CI 0.97, 1.6] and 1.1 nM [95% CI 0.94, 1.6] for imidacloprid and thiacloprid respectively for honeybees, and IC_{50} of 0.71 [95% CI 0.62, 0.82] and 0.62 nM [95% CI 0.50, 0.77] for imidacloprid and thiacloprid for bumble bees). This finding clearly demonstrates that the differences in bee sensitivity to these two neonicotinoids is not a consequence of variation in their affinity for the nAChR.

The use of insecticide synergists that inhibit insect metabolic enzyme systems has provided evidence that one or more members of the cytochrome P450 superfamily are responsible for the tolerance of honeybees to thiacloprid [2]. Indeed, Iwasa et al. [2] demonstrated that the P450 inhibitors piperonyl butoxide (PBO), triflumizole, and propiconazole increased honeybee sensitivity to thiacloprid by 154-, 1,141- and 559-fold, respectively, but had almost no effect on honeybee sensitivity to imidacloprid. To explore this further, we used 1-aminobenzotriazole (ABT), a nonspecific suicide inhibitor of P450s that has been used widely in mammalian systems to distinguish P450-mediated metabolism from non-P450-mediated metabolism *in vitro* [11, 12]. Honeybees pretreated with ABT became > 170-fold more sensitive to thiacloprid but only 2.7-fold more sensitive to imidacloprid (Figure 1C), supporting the view that P450s underlie the variation in the sensitivity of this bee species to these two compounds. Likewise, insecticide bioassays of bumble bees after treatment with PBO resulted in a significant 4.2-fold increase in the sensitivity of bumble bees to thiacloprid but no significant shift in sensitivity to imidacloprid (Table S1). Thus, we demonstrate that P450s also appear to be an important determinant of neonicotinoid sensitivity in bumble bees. The level of synergism we observed in bumble bees is significantly lower than that reported by Iwasa et al. [2] using the same inhibitor (see above); this may in part result from differences in methodology used (contact versus oral insecticide bioassays) and/or differences in the ability of this synergist to inhibit the relevant P450 enzymes.

Insect P450 genes fall into four major clades, and enzymes from each of these clades have been linked to insecticide resistance or to the metabolism of xenobiotics [13]. However, members of the CYP3 clade, particularly those of the CYP6 and CYP9 families, have been most frequently linked to xenobiotic detoxification across a range of insect species [13–15]. Therefore, to explore which honeybee P450(s) are responsible for thiacloprid metabolism, 27 of the 46 honeybee P450 genes, comprising the entire CYP3 clade, were individually coexpressed with house fly P450 reductase (CPR) in an insect cell line. Incubation of purified microsomal preparations containing each P450 and CPR with thiacloprid and analysis of the metabolites produced by liquid chromatography tandem mass spectrometry (LC-MS/MS) identified a single P450, CYP9Q3, as the highly efficient metabolizer of thiacloprid (primarily to 5-hydroxy thiacloprid) (Figures 2A, S1, and S2). Topical bioassays of honeybees using 5-hydroxy thiacloprid revealed reduced toxicity of this metabolite (LD_{50}-value of > 100 µg/bee) relative to the parent compound (Figure 1A). Three other P450s—CYP6AS5, CYP9Q1, and CYP9Q2—showed weak activity against thiacloprid, but this was at least > 10-fold lower than that seen for CYP9Q3 (Figure 2A). Repeating these assays using imidacloprid revealed that only CYP9Q1–3 exhibit any capacity to metabolize this compound but at much lower efficiency than exhibited for thiacloprid (Figure 2A). To provide additional evidence that CYP9Q3 is the primary honeybee P450 that confers tolerance to thiacloprid *in vivo*, we created a series of transgenic *Drosophila* lines expressing honeybee *CYP9Q1*, *CYP9Q2*, or *CYP9Q3*. Flies expressing the *CYP9Q3* transgene showed a marked (> 10-fold) and significant resistance to thiacloprid compared to control flies of the same genetic background without the transgene in insecticide bioassays (Figure 2D). Flies expressing *CYP9Q1* showed no change in sensitivity to thiacloprid compared to controls, and flies expressing *CYP9Q2* showed a significant but more modest (3.5-fold) resistance to thiacloprid (Figure 2D). In bioassays using imidacloprid, no significant differences in sensitivity were observed between flies with any of the three transgenes and control flies consistent with the low efficiency of imidacloprid

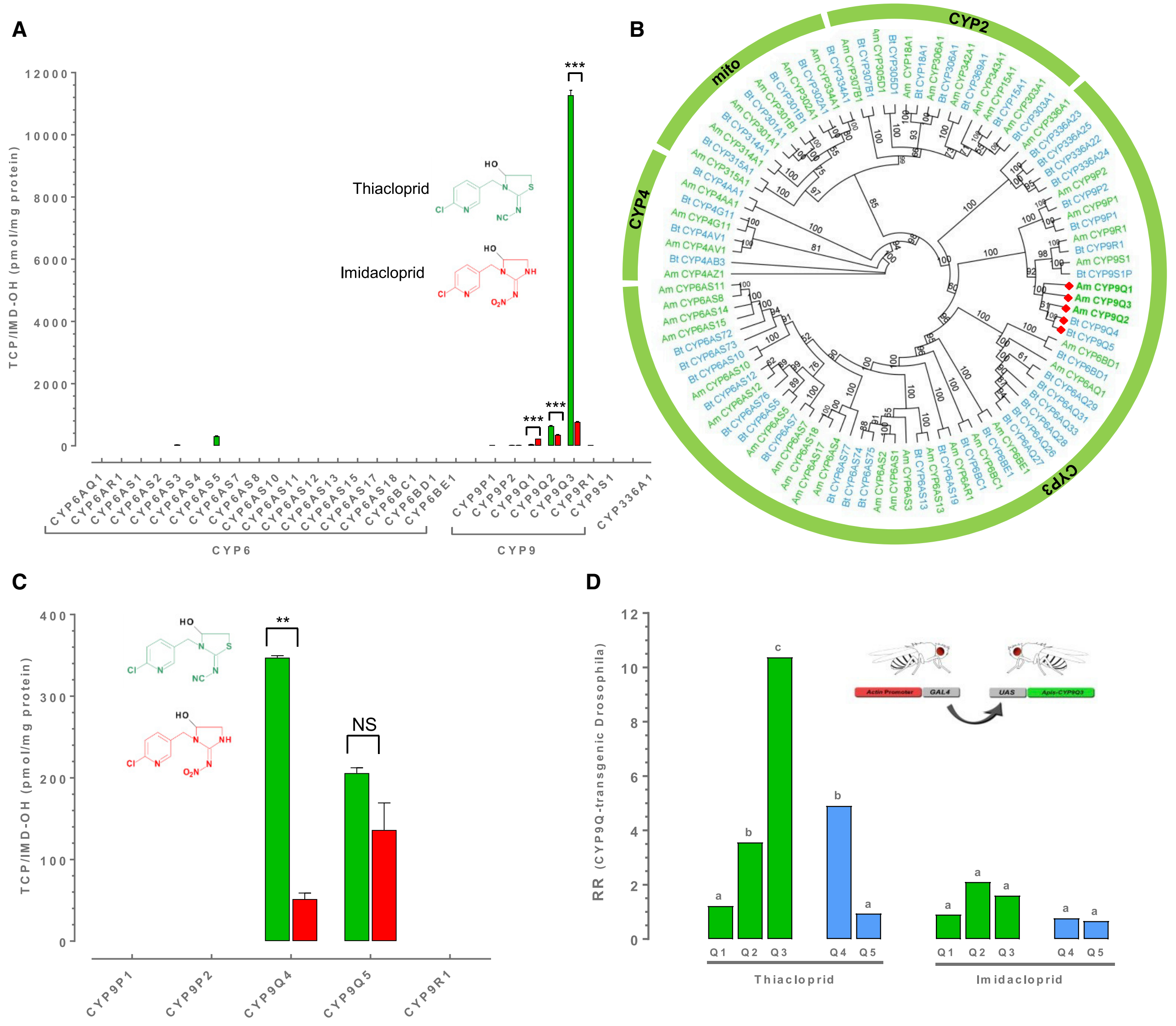

Figure 2. Identification of Neonicotinoid Metabolising P450s in Honeybee and Bumble Bee

(A and C) (A) Thiacloprid and imidacloprid hydroxylation by recombinantly expressed P450s of the *A. mellifera* CYP3 clade and (C) the CYP9 family in *B. terrestris*. The production of the hydroxy metabolite of each insecticide is displayed per mg of P450 protein (NS, not significant; **Pc < c0.01, ***Pc < c0.001; Welch's t test). Error bars display standard deviation (n = 3).

(B) Phylogenetic tree with branch bootstrap values for *A. mellifera* (green) and *B. terrestris* (blue) P450 genes. Genes are grouped according to their adscription to different P450 clades. Branches within the CYP3 clade marked with a red dot indicate the position of *A. mellifera* CYP9Qs and their closest *B. terrestris* orthologs involved in thiacloprid metabolism, as shown in (A), (C), and (D).

(D) Resistance ratio (RR) of transgenic *Drosophila* strains expressing *A. mellifera AmCYP9Q1–3* or *B. terrestris BtCYP9Q4-5* transgenes to thiacloprid and imidacloprid compared to a control line (flies of the same genetic background but without the transgene). Significance is referenced against this control line and based on non-overlapping 95% fiducial limits of LC_{50} values (n = 3).

See also Figures S1, S2, and S3.

metabolism observed *in vitro* (Figure 2D). Taken together, these results demonstrate unequivocally that the transcription of *CYP9Q3* confers strong intrinsic tolerance to thiacloprid, but not to imidacloprid.

To identify potential functional orthologs of honeybee *CYP9Q3* in the bumble bee, we compared P450s identified in the sequenced genome of *B. terrestris* [16] with *CYP9Q1–3*. Phylogenetic analysis of the *B. terrestris* CYPome revealed five candidate genes subsequently named as *CYP9P1*, *CYP9P2*, *CYP9R1*, *CYP9Q4*, and *CYP9Q5* that cluster with honeybee *CYP9Q1–3* (Figure 2B). Of these, *CYP9Q4* and *CYP9Q5* show the greatest sequence identity to honeybee *CYP9Q1–3* (Figure S3). Functional expression of these five P450s *in vitro* revealed that only CYP9Q4 and CYP9Q5 metabolize thiacloprid to its 5-hydroxy form (Figure 2C), with subsequent enzyme kinetic assays confirming that CYP9Q4 metabolizes thiacloprid more efficiently

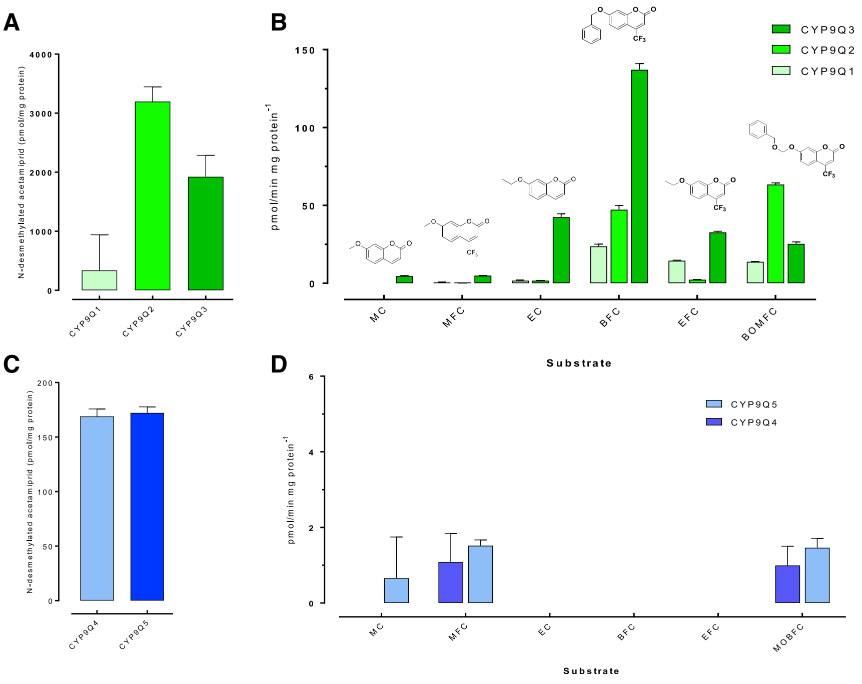

Figure 3. Metabolism of Acetamiprid and Model Substrates by Honeybee and Bumble Bee CYP9Q Subfamily P450s

(A and C) Acetamiprid N-desmethylation by recombinantly expressed CYP9Q1–3 of *A. mellifera* and (C) CYP9Q4–5 of *B. terrestris*. The production of N-desmethylated acetamiprid is displayed per mg of protein. Error bars display standard deviation (n = 3).

(B and D) (B) Activity of CYP9Q1–3 and (D) CYP9Q4–5 against different fluorescent coumarin model substrates. Error bars display standard deviation (n = 3). Abbreviations: MC, 7-methoxycoumarin; MFC, 7-methoxy-4-trifluoromethyl coumarin; EC, 7-ethoxy coumarin; BFC, 7-benzyloxy-4-trifluoromethyl coumarin; EFC, 7-ethoxy-4-trifluoromethyl coumarin; BOMFC, 7-benzyloxymethoxy-4-trifluoromethyl coumarin; MOBFC, 7-p-methoxy-benzyloxy-4-trifluoro coumarin.

than CYP9Q5 (Figure S2). Further functional validation of these two P450s by expression in transgenic *Drosophila* revealed that flies expressing *CYP9Q4* exhibited significant (~5-fold) resistance to thiacloprid compared to controls, whereas flies expressing *CYP9Q5* showed no change in sensitivity (Figure 2D). As for honeybee *CYP9Q1–3*, no significant differences were observed in the sensitivity of flies expressing either *CYP9Q4* or *CYP9Q5* to imidacloprid compared to controls (Figure 2D). Thus, these findings demonstrate that members of the *CYP9Q* subfamily also confer tolerance to thiacloprid in *B. terrestris*.

To further explore the substrate specificity of CYP9Q1–5, we tested their functional activity against a range of fluorescent model substrates and acetamiprid, a second N-cyanoamidine neonicotinoid that also has low acute toxicity to honeybees and is very rapidly metabolized *in vivo* [4]. Against coumarin model substrates, honeybee CYP9Q1–3 show a preference for bulkier molecules such as BFC and BOMFC, with CYP9Q1 and CYP9Q3 both showing highest specific activity for BFC (Figure 3B). In addition, CYP9Q3 demonstrated a pattern of broader substrate specificity than the other two P450s, suggestive of a more promiscuous active site (Figure 3B). These results contrasted with bumble bee CYP9Q4 and CYP9Q5, which showed no activity against BFC and, in the case of CYP9Q4, a noticeably reduced substrate specificity with activity against just two of the model substrates tested (MFC and MOBFC) (Figure 3D). Incubation of recombinant CYP9Q1–5 with acetamiprid followed by LC-MS/MS analyses revealed that all five P450s have the capacity to metabolize this compound to N-desmethyl acetamiprid, with CYP9Q2–5 exhibiting the highest activity (Figures 3A and 3C). Thus, our data demonstrate that the rapid metabolism of acetamiprid reported *in vivo* [4] is likely mediated, at least in part, by P450s of the CYP9Q subfamily.

The CYP9Q subfamily of P450s has been implicated in the metabolism of xenobiotics previously, with honeybee CYP9Q1–3 all found to metabolize the pyrethroid tau-fluvalinate and the organophosphate coumaphos, two insecticides that show marked selectivity for mites (i.e., *Varroa*) over bees [15]. Our findings reveal that in both honey bees and bumble bees, this P450 subfamily contains potent metabolizers of certain neonicotinoid insecticides, thus explaining the low acute toxicity of thiacloprid and acetamiprid. In humans, just a handful of the 57 functional P450s are responsible for the biotransformation of most foreign chemicals; for example, CYP3A4 and CYP2D6 together are responsible for the metabolism of > 50% of clinically used drugs [17]. The finding that members of the bee CYP9Q subfamily have the capacity to metabolize compounds belonging to three different insecticide classes suggests that they may act as functional insect equivalents of these human P450s and thus are critically important in defining the sensitivity of eusocial bees to xenobiotics.

To identify the primary sites of CYP9Q-mediated detoxification, P450 expression was assessed in bee body parts and dissected tissues that are commonly involved in xenobiotic detoxification [18] by quantitative PCR (qPCR). *CYP9Q3* was expressed at high levels in the honeybee brain and Malpighian tubules (Figure 4A), the latter finding consistent with a previous study which examined expression in honeybee tissues, including the Malpighian tubules, by RNA-seq [19]. In *B. terrestris*, *CYP9Q4* and *CYP9Q5* showed marked differences in their pattern of spatial expression, with *CYP9Q4* highly expressed in the brain (> 60-fold greater than in the other tissues tested) and *CYP9Q5* expressed at relatively uniform levels in the midgut, Malpighian tubules, and brain (Figure 4A). To examine the expression of *CYP9Q3* at higher resolution, we used *in situ* hybridization with digoxigenin-labeled RNA probes to localize *CYP9Q3* expression in the brain and Malpighian tubules. This revealed that *CYP9Q3* is expressed at particularly high levels in the proximal regions of Malpighian tubules and where they join the midgut-hindgut junction and in several structures of the honeybee brain, including the optic and antennal lobes and the mushroom bodies (Figures 4B and 4C). Malpighian tubules are the functional insect equivalents of vertebrate kidneys, and these

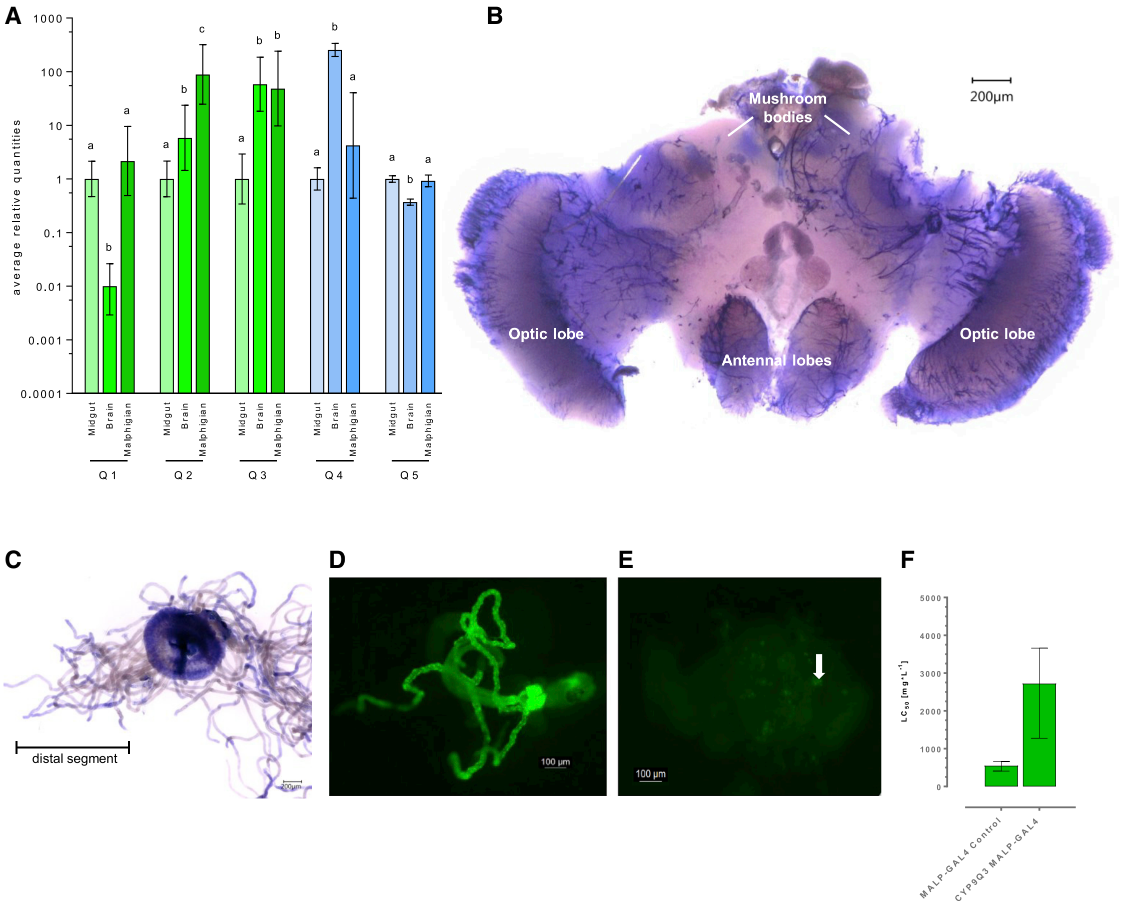

Figure 4. Tissue-Specific Expression and Functional Characterization of Honeybee and Bumble Bee Neonicotinoid-Metabolizing P450s

(A) Relative expression (fold change) of *A. mellifera* and *B. terrestris* thiacloprid metabolising CYP9Q genes in different tissues of worker bees measured by qPCR. Significant differences ($p < 0.01$) in expression between tissues is denoted using letters above bars as determined by One-Way ANOVA with post hoc testing (Benjamini and Hochberg).

(B and C) (B) Whole-mount *in situ* hybridization showing the distribution and abundance of the *AmCYP9Q3* transcript in the brain of a worker bee in different neuronal cells and in (C) the Malpighian tubules and distal midgut.

(D and E) Expression of green fluorescent protein in the Malpighian tubules and specific neurons of the *Drosophila* brain driven by the Malp-tub GAL4 line.

(F) Sensitivity of transgenic *Drosophila* to thiacloprid when the Malp-tub GAL4 line is used to drive expression of *AmCYP9Q3.* Error bars display 95% CLs.

osmoregulatory and detoxifying organs absorb solutes, water, and wastes from the surrounding haemolymph. The high expression of *CYP9Q3* in this tissue is therefore highly consistent with a primary role in xenobiotic detoxification. The expression of *CYP9Q3* and especially *CYP9Q4* in the bee brain suggests a secondary or additional site of detoxification against xenobiotics that cross the blood-brain barrier, and it is notable that the structures of the brain expressing *CYP9Q3* have been previously highlighted as sites of AChE activity and nAChR-like immunoreactivity [20]. Based on this finding, we explored the effect of specifically expressing *CYP9Q3* in the Malpighian tubules and the insect brain on sensitivity to thiacloprid by exploiting the GAL4/UAS system of *Drosophila*. Significant levels of thiacloprid resistance were observed in transgenic *Drosophila* when expression of *CYP9Q3* was directed to the Malpighian tubules and neuronal cells (ellipsoid body, pars intercerebralis, fan-shaped and large-field neurons) (Figures 4D and 4E and 4F), demonstrating that expression of *CYP9Q3* at these sites is sufficient to provide protection against this insecticide. Previous studies have examined the expression of honeybee *CYP9Q* P450s in different life stages of bees. For example, a recent study performed RNA-seq of different tissues in honeybee foragers, older workers which gather and process food, and nurses, young workers that care for brood [19]. While no change was observed in *CYP9Q3* expression in the Malpighian tubules and midgut between the two worker roles, foragers showed higher levels of expression in the mandibular and hypopharyngeal glands [19]. These findings were consistent with a second study, which examined the expression of *CYP9Q1–3* in the legs and antennae of newly eclosed workers, nurses, and foragers and observed a pattern of increased expression with age [21]. The greater expression of these P450s in foragers is consistent with their increased exposure to xenobiotics compared to nurses, and their elevated expression in tissues that mediate nectar processing suggests that they may provide a first line of defense against dietary xenobiotics.

Sequencing of the honeybee genome and the discovery that it contains a reduced number of genes encoding detoxification

enzymes (including P450s) led to the suggestion that bees may be particularly sensitive to xenobiotics, including pesticides [22]. However, a subsequent meta-analysis of available toxicological data revealed that honeybees are, in fact, no more sensitive to insecticides than other insect species [23]. Both honeybees and bumble bees have undergone millions of years of selection to evolve mechanisms to overcome the diverse array of toxic compounds that occur naturally in their environment [6]. Although this does not include the relatively recently introduced synthetic insecticides, our study, in combination with previous work [15], demonstrates that these existing detoxification pathways can be recruited to protect bees from pesticides if sufficient similarity exists between their native substrate(s) and the synthetic compound in question. In this regard, although the diversity of native substrates that the CYP9Q subfamily can metabolize is not fully understood, all members of this subfamily in honeybees have been shown to metabolize the plant secondary metabolite quercetin with high efficiency, a flavonoid that is present in pollen and nectar, which inhibits mitochondrial ATP synthase [15].

In conclusion, these data demonstrate that the CYP9Q family of both honeybees and bumble bees contains critically important enzymes that define their sensitivity to neonicotinoids. This finding illustrates the importance of considering bee xenobiotic biotransformation pathways to predict, and potentially influence, the pharmacological and toxicological outcomes of insecticide use. For example, the knowledge and tools developed in this study can be harnessed to avoid negative pesticide-pesticide interactions [24] due to inhibition of these key defense systems. Furthermore, our findings, and those of previous studies that have uncovered the molecular and biochemical basis of pesticide selectivity [15, 25–29], can facilitate the development of compounds that show high efficacy against crop pests but low toxicity to nontarget beneficial insects. In this regard, the recombinant enzymes and transgenic *Drosophila* lines developed in our study can be used as screening tools to assess the metabolic liability of future insecticidal lead compounds and so ensure that they are rapidly broken down by these major xenobiotic detoxifying enzymes.

STAR★METHODS

Detailed methods are provided in the online version of this paper and include the following:

- KEY RESOURCES TABLE
- CONTACT FOR REAGENT AND RESOURCE SHARING
- EXPERIMENTAL MODEL AND SUBJECT DETAILS
  - Insects
  - Insect cell lines
- METHOD DETAILS
  - Insecticide bioassays of *A. mellifera* and *B. terrestris*
  - Receptor binding studies
  - Functional expression of bee P450s
  - Metabolism assays and UPLC-MS/MS analysis
  - Functional activity of recombinant P450s against fluorescent model substrates
  - Transgenic expression of bee P450s in *D. melanogaster*
  - Expression profiling of bee P450s
- QUANTIFICATION AND STATISTICAL ANALYSIS
- DATA AND SOFTWARE AVAILABILITY

SUPPLEMENTAL INFORMATION

Supplemental Information includes three figures and three tables and can be found with this article online at https://doi.org/10.1016/j.cub.2018.02.045.

ACKNOWLEDGMENTS

We thank David Nelson (Department of Molecular Science, University of Tennessee) for naming the P450s identified in this study. We thank Christian Maus (Bayer Bee Care Center) for his comments on an earlier draft of the paper. This study received funding from Bayer AG. C.B. received funding from the European Research Council (ERC) under the European Union's Horizon 2020 research and innovation programme (grant agreement no. 646625). C.B. and K.B. received funding from Biotechnology and Biological Sciences Research Council (BBSRC, award number 15076182). The work at Rothamsted forms part of the Smart Crop Protection (SCP) strategic programme (BBS/OS/CP/000001) funded through the Biotechnology and Biological Sciences Research Council's Industrial Strategy Challenge Fund.

AUTHOR CONTRIBUTIONS

C.M., B.J.T., M.Z., K.B., E.R., G.H., K.S.S., C.T.Z., R.A.H., B.L., R.R., L.K., M.K., C.B., and R.N. performed experiments and analysis. C.B. and R.N. conceived and designed the study and drafted the manuscript. C.B., R.N., J.B., M.S.W., T.G.E.D., and L.M.F. directed the study. All authors helped write the manuscript.

DECLARATION OF INTERESTS

This study received funding from Bayer AG, a manufacturer of neonicotinoid insecticides.

Received: January 24, 2018
Revised: February 15, 2018
Accepted: February 16, 2018
Published: March 22, 2018

REFERENCES

1. Cressey, D. (2017). The bitter battle over the world's most popular insecticides. Nature *551*, 156–158.
2. Iwasa, T., Motoyama, N., Ambrose, J.T., and Roe, M. (2004). Mechanism for the differential toxicity of neonicotinoid insecticides in the honey bee, *Apis mellifera*. Crop Prot. *23*, 371–378.
3. Alptekin, S., Bass, C., Nicholls, C., Paine, M.J., Clark, S.J., Field, L., and Moores, G.D. (2016). Induced thiacloprid insensitivity in honeybees (*Apis mellifera* L.) is associated with up-regulation of detoxification genes. Insect Mol. Biol. *25*, 171–180.
4. Brunet, J.L., Badiou, A., and Belzunces, L.P. (2005). *In vivo* metabolic fate of [14C]-acetamiprid in six biological compartments of the honeybee, *Apis mellifera* L. Pest Manag. Sci. *61*, 742–748.
5. Suchail, S., De Sousa, G., Rahmani, R., and Belzunces, L.P. (2004). *In vivo* distribution and metabolisation of 14C-imidacloprid in different compartments of *Apis mellifera* L. Pest Manag. Sci. *60*, 1056–1062.
6. Johnson, R.M. (2015). Honey bee toxicology. Annu. Rev. Entomol. *60*, 415–434.
7. Berenbaum, M.R., and Johnson, R.M. (2015). Xenobiotic detoxification pathways in honey bees. Curr. Opin. Insect Sci. *10*, 51–58.
8. Agency, U.S.E.P. (2014). Guidance for assessing pesticide risks to bees. https://www.epa.gov/sites/production/files/2014-06/documents/pollinator_risk_assessment_guidance_06_19_14.pdf.
9. Nauen, R., Ebbinghaus-Kintscher, U., and Schmuck, R. (2001). Toxicity and nicotinic acetylcholine receptor interaction of imidacloprid and its

metabolites in *Apis mellifera* (Hymenoptera: Apidae). Pest Manag. Sci. *57*, 577–586.

10. Schmuck, R. (2001). Ecotoxicological profile of the insecticide thiacloprid. Pflanzenschutz Nachrichten-Bayer-English Edition *54*, 161–184.
11. Kenneke, J.F., Mazur, C.S., Ritger, S.E., and Sack, T.J. (2008). Mechanistic investigation of the noncytochrome P450-mediated metabolism of triadimefon to triadimenol in hepatic microsomes. Chem. Res. Toxicol. *21*, 1997–2004.
12. Wang, M.Z., Saulter, J.Y., Usuki, E., Cheung, Y.L., Hall, M., Bridges, A.S., Loewen, G., Parkinson, O.T., Stephens, C.E., Allen, J.L., et al. (2006). CYP4F enzymes are the major enzymes in human liver microsomes that catalyze the O-demethylation of the antiparasitic prodrug DB289 [2,5-bis(4-amidinophenyl)furan-bis-O-methylamidoxime]. Drug Metab. Dispos. *34*, 1985–1994.
13. Feyereisen, R. (2006). Evolution of insect P450. Biochem. Soc. Trans. *34*, 1252–1255.
14. Li, X., Schuler, M.A., and Berenbaum, M.R. (2007). Molecular mechanisms of metabolic resistance to synthetic and natural xenobiotics. Annu. Rev. Entomol. *52*, 231–253.
15. Mao, W., Schuler, M.A., and Berenbaum, M.R. (2011). CYP9Q-mediated detoxification of acaricides in the honey bee (*Apis mellifera*). Proc. Natl. Acad. Sci. USA *108*, 12657–12662.
16. Sadd, B.M., Barribeau, S.M., Bloch, G., de Graaf, D.C., Dearden, P., Elsik, C.G., Gadau, J., Grimmelikhuijzen, C.J.P., Hasselmann, M., Lozier, J.D., et al. (2015). The genomes of two key bumblebee species with primitive eusocial organization. Genome Biol. *16*, 76.
17. Zanger, U.M., and Schwab, M. (2013). Cytochrome P450 enzymes in drug metabolism: regulation of gene expression, enzyme activities, and impact of genetic variation. Pharmacol. Ther. *138*, 103–141.
18. Zhu, F., Parthasarathy, R., Bai, H., Woithe, K., Kaussmann, M., Nauen, R., Harrison, D.A., and Palli, S.R. (2010). A brain-specific cytochrome P450 responsible for the majority of deltamethrin resistance in the QTC279 strain of *Tribolium castaneum*. Proc. Natl. Acad. Sci. USA *107*, 8557–8562.
19. Vannette, R.L., Mohamed, A., and Johnson, B.R. (2015). Forager bees (*Apis mellifera*) highly express immune and detoxification genes in tissues associated with nectar processing. Sci. Rep. *5*, 16224.
20. Gauthier, M. (2010). State of the art on insect nicotinic acetylcholine receptor function in learning and memory. Adv. Exp. Med. Biol. *683*, 97–115.
21. Mao, W., Schuler, M.A., and Berenbaum, M.R. (2015). Task-related differential expression of four cytochrome P450 genes in honeybee appendages. Insect Mol. Biol. *24*, 582–588.
22. Claudianos, C., Ranson, H., Johnson, R.M., Biswas, S., Schuler, M.A., Berenbaum, M.R., Feyereisen, R., and Oakeshott, J.G. (2006). A deficit of detoxification enzymes: pesticide sensitivity and environmental response in the honeybee. Insect Mol. Biol. *15*, 615–636.
23. Hardstone, M.C., and Scott, J.G. (2010). Is *Apis mellifera* more sensitive to insecticides than other insects? Pest Manag. Sci. *66*, 1171–1180.
24. Johnson, R.M., Dahlgren, L., Siegfried, B.D., and Ellis, M.D. (2013). Acaricide, fungicide and drug interactions in honey bees (*Apis mellifera*). PLoS ONE *8*, e54092.
25. Andrews, M.C., Callaghan, A., Field, L.M., Williamson, M.S., and Moores, G.D. (2004). Identification of mutations conferring insecticide-insensitive AChE in the cotton-melon aphid, *Aphis gossypii* Glover. Insect Mol. Biol. *13*, 555–561.
26. Bass, C., Puinean, A.M., Andrews, M., Cutler, P., Daniels, M., Elias, J., Paul, V.L., Crossthwaite, A.J., Denholm, I., Field, L.M., et al. (2011). Mutation of a nicotinic acetylcholine receptor β subunit is associated with resistance to neonicotinoid insecticides in the aphid *Myzus persicae*. BMC Neurosci. *12*, 51.
27. Bravo, A., Gómez, I., Porta, H., García-Gómez, B.I., Rodriguez-Almazan, C., Pardo, L., and Soberón, M. (2013). Evolution of *Bacillus thuringiensis* Cry toxins insecticidal activity. Microb. Biotechnol. *6*, 17–26.
28. Shimomura, M., Yokota, M., Ihara, M., Akamatsu, M., Sattelle, D.B., and Matsuda, K. (2006). Role in the selectivity of neonicotinoids of insect-specific basic residues in loop D of the nicotinic acetylcholine receptor agonist binding site. Mol. Pharmacol. *70*, 1255–1263.
29. Wu, S., Nomura, Y., Du, Y., Zhorov, B.S., and Dong, K. (2017). Molecular basis of selective resistance of the bumblebee $BiNa_v1$ sodium channel to tau-fluvalinate. Proc. Natl. Acad. Sci. USA *114*, 12922–12927.
30. OECD (1998). Guideline for the testing of chemicals. Honeybees, acute oral toxicity test. OECD 213. (doi:https://doi.org/10.1787/9789264070165-en).
31. Schenkman, J.B., and Jansson, I. (2006). Spectral Analyses of Cytochromes P450. In Cytochrome P450 Protocols, I.R. Phillips, and E.A. Shephard, eds. (Humana Press), pp. 11–18.
32. Markstein, M., Pitsouli, C., Villalta, C., Celniker, S.E., and Perrimon, N. (2008). Exploiting position effects and the gypsy retrovirus insulator to engineer precisely expressed transgenes. Nat. Genet. *40*, 476–483.
33. Riveron, J.M., Irving, H., Ndula, M., Barnes, K.G., Ibrahim, S.S., Paine, M.J., and Wondji, C.S. (2013). Directionally selected cytochrome P450 alleles are driving the spread of pyrethroid resistance in the major malaria vector *Anopheles funestus*. Proc. Natl. Acad. Sci. USA *110*, 252–257.
34. Pfaffl, M.W. (2001). A new mathematical model for relative quantification in real-time RT-PCR. Nucleic Acids Res. *29*, e45.
35. Horňáková, D., Matousková, P., Kindl, J., Valterová, I., and Pichová, I. (2010). Selection of reference genes for real-time polymerase chain reaction analysis in tissues from *Bombus terrestris* and *Bombus lucorum* of different ages. Anal. Biochem. *397*, 118–120.
36. Lourenço, A.P., Mackert, A., dos Santos Cristino, A., and Simões, Z.L.P. (2008). Validation of reference genes for gene expression studies in the honey bee, *Apis mellifera*, by quantitative real-time RT-PCR. Apidologie (Celle) *39*, 372–385.
37. Reim, T., Thamm, M., Rolke, D., Blenau, W., and Scheiner, R. (2013). Suitability of three common reference genes for quantitative real-time PCR in honey bees. Apidologie (Celle) *44*, 342–350.

STAR★METHODS

KEY RESOURCES TABLE

REAGENT or RESOURCE	SOURCE	IDENTIFIER
Antibodies		
Alkaline phosphatase labeled antidigoxygenin antibody	abcam	Cat# ab6212
Biological Samples		
Bumblebee Colony	Agralan UK Ltd	Cat# M644
Chemicals, Peptides, and Recombinant Proteins		
NutriFly premix food	SLS	Cat# FLY1034
Phusion HF DNA polymerase	Thermo Fisher	Cat# 10024537
SYBR Green JumpStart Taq Readymix	Sigma-Aldrich	Cat# S4438500RXN
Bradford reagent	Sigma-Aldrich	Cat# B6916-500ML
NADPH	Sigma-Aldrich	Cat# N1630-25MG
Glutathione oxidized	Sigma-Aldrich	Cat# 64501
Glutathione reductase	Sigma-Aldrich	Cat# G3664
7-Hydroxycoumarin (HC)	Sigma-Aldrich	Cat# 202-240-3
7-Hydroxy-4-(trifluoromethyl)coumarin (HFC)	Sigma-Aldrich	Cat# 368512-250MG
7-methoxy-coumarin (MC)	Sigma-Aldrich	Cat# W515809-25G
7-Methoxy-4-(tri-fluoromethyl)-coumarin (MFC)	Sigma-Aldrich	Cat# T3165-100MG
7-ethoxy-coumarin (EC)	Sigma-Aldrich	Cat# E1379-100MG
7-benzyloxy-4-(trifluoromethyl)-coumarin (BFC)	Sigma-Aldrich	Cat# 5057-5MG
7-ethoxy-4-trifluoro-methylcoumarin (EFC)	Sigma-Aldrich	Cat# 46127-100MG
7-benzyloxymethoxy-4-trifluoromethyl coumarin (BOMFC)	Sigma-Aldrich	Cat# 5047-5MG
Bovine Serum Albumin (BSA)	Sigma-Aldrich	Cat# P0834-10X1ML
Piperonyl butoxide (PBO)	Sigma-Aldrich	Cat# 291102-100ML
Pollen	Sussex Wholefoods	Cat# 7BEP2
Critical Commercial Assays		
ISOLATE II RNA Mini Kit	Bioline	Cat# BIO-52073
SuperScript III Reverse Transcriptase kit	Invitrogen	Cat# 18080044
Imidacloprid	Bayer CropScience	n/a
Thiacloprid	Bayer CropScience	n/a
Acetamiprid	Bayer CropScience	n/a
Bac-to-Bac Baculovirus Expression System	GIBCO	Cat# 10359016
NADPH Regeneration system	Promega	Cat# V9510
SsoAdvanced Universal SYBR® Green Supermix	BIO-RAD	Cat# 1725271
PicoPure RNA Isolation Kit	Thermo Fisher	Cat# KIT0204
iScript cDNA Synthesis Kit	BIO-RAD	Cat# 1708891
Plant DNeasy Mini Kit	QIAGEN	Cat# 69104
Deposited Data		
See Table S3 for accession numbers of P450s characterized in this study	N/A	See Table S3
Experimental Models: Cell Lines		
Sf9	GIBCO	Cat# 11496015
High Five	GIBCO	Cat# B85502

(*Continued on next page*)

Continued		
REAGENT or RESOURCE	SOURCE	IDENTIFIER
Experimental Models: Organisms/Strains		
Drosophila melanogaster:13-20: "y^1w^{67c23}; P attP40 25C6," "1;2"	University of Cambridge	Stock 13-20
Drosophila melanogaster: Act5C-GAL4: ["y[1] w[*]; P(Act5C-GAL4-w)E1/CyO,""1;2"	Bloomington Stock Center	Cat# 25374
Drosophila melanogaster: *Malp-GAL4*: *w[*]; P{w[+mW.hs] = GawB}c42*	Bloomington Stock Center	Cat# 30835
Drosophila melanogaster: *UAS-GFP*: w^{1118}; $P\{w^{+mC}$ = *UAS-GFP.nls}14*	Bloomington Stock Center	Cat# 4775
Oligonucleotides		
See Supplemental Materials	N/A	See Table S2
Recombinant DNA		
Cytochrome P450 variants	GeneArt, CA, USA	See Table S3
Cytochrome P450 reductase (CPR)	GeneArt, CA, USA	GenBank: Q07994
pUASTattB40 Vector	Gift from Jacob Riveron, Liverpool School of Tropical Medicine	GenBank: EF362409.1
Gateway pDEST8 expression vector	Invitrogen	Cat# 11804010
Software and Algorithms		
Geneious v 9.1.8	Biomatters	https://www.geneious.com/download/
Genstat v 16	VSN International	https://www.vsni.co.uk/software/genstat/
SoftMax Pro 7	Molecular devices	https://www.moleculardevices.com/systems/microplate-readers/softmax-pro-7-software
GraphPad Prism v 7	GraphPad Software Inc.	https://www.graphpad.com/
SpectralWorks	SpectralWorks Ltd	https://www.spectralworks.com/
$qbase^+$ v 3.1	Biogazelle	https://www.qbaseplus.com/

CONTACT FOR REAGENT AND RESOURCE SHARING

Further information and requests may be directed to and will be fulfilled by the Lead Contact, Chris Bass (chris.bass@exeter.ac.uk).

EXPERIMENTAL MODEL AND SUBJECT DETAILS

Insects

Adult honeybees were obtained from open hives during the summer of 2014-2016 that were maintained pesticide-free by bee keepers at Bayer AG, CropScience Division, Monheim, Germany. Bumblebee colonies were purchased from Agralan UK Ltd and maintained in constant darkness at 25°C, 50% RH. The colonies were fed *ad libitum* on the nectar substitute, Biogluc®, and pollen was supplied to colonies every 2 days.

The *Drosophila melanogaster* stock 13-20 ["y^1w^{67c23}; P attP40 25C6," "1;2"] obtained from the University of Cambridge was used to create all transgenic lines. Virgin females of this line were crossed to males of the Act5C-GAL4 strain ["y[1] w[*]; P(Act5C-GAL4-w) E1/CyO,""1;2"] (Bloomington Stock Center) to activate transgene expression (see below for description of methods). The *Malp-GAL4* strain [*w[*]; P{w[+mW.hs] = GawB}c42*] (Bloomington Stock Center) which expresses *GAL4* in the Malpighian tubules and specific neuronal cells (ellipsoid body, pars intercerebralis, fan shaped and large field neurons), was used to drive the expression of *CYP9Q3* in these tissues. The *UAS-GFP* strain [w^{1118}; $P\{w^{+mC}$ = *UAS-GFP.nls}14*] (Bloomington Stock Center) was used to visualize the sites of expression driven by *Act5C-GAL4* and *Malp-GAL4* drivers. All flies were reared on NutriFly food (NLS) at 24°C. Only female flies 2-5 days post eclosion were used for insecticide bioassays.

Insect cell lines

The Sf9 and High Five insect cell lines (ovarian cells from *Spodoptera frugiperda* and *Trichoplusia ni* respectively) were maintained in suspension culture under serum-free conditions at 27°C containing 25 μg/ml^{-1} gentamycin in SF-900 II SFM (GIBCO) and Express Five SFM (GIBCO), respectively.

METHOD DETAILS

Insecticide bioassays of *A. mellifera* and *B. terrestris*

Acute contact insecticide assays were performed on female *A. mellifera* following standard methods OECD 2013 [30]. Bioassays of *B. terrestris* were based on the OECD guidelines developed for honeybees [30] but with bees assayed in individual Nicot cages. Bees were starved of sucrose solution for up to 2 hr to encourage feeding during the experiment. Individual *B. terrestris* were fed with 20 ul of insecticide-sucrose solution at concentrations of 0.01, 0.1, 1, 10 and 100 ppm for imidacloprid and 10, 50, 100, 500 and 1000 ppm for thiacloprid. Controls were fed a solution of sucrose containing a concentration of acetone matching that of the highest treatment concentration. After 4-6 hr the syringes were assessed to see if bees had consumed the insecticide-sucrose solution. Those that had not consumed all of the solution were excluded from the experiment. Mortality was assessed 48 hr after feeding and lethal concentrations (LC_{50} values) were calculated by probit analysis using Genstat version 16 (VSN International). For synergist bioassays, *B. terrestris* or *A. mellifera* workers were first treated with 20 μg of piperonyl butoxide or 1 μg of aminobenzotriazole applied to the dorsal thorax. Synergist bioassays included an additional control group treated only with the synergist. 1 hr after synergist application, bees were then treated with the appropriate insecticide dosage as above.

Receptor binding studies

[^{3}H]imidacloprid (specific activity 1.406 GBq μmol^{-1}) displacement studies were conducted using membrane preparations isolated from frozen (−80°C) honeybee and bumble bee heads, respectively, following previously published protocols [9]. Briefly, bee heads weighing 10cg were homogenized in 200cml ice-cold 0.1cM potassium phosphate buffer, pH 7.4 containing 95cmM sucrose using a motor-driven Ultra Turrax blender. The homogenate was then centrifuged for 10cmin at 1200cg and the resulting supernatant filtered through five layers of cheesecloth with protein concentration determined using Bradford reagent (Sigma) and bovine serum albumin (BSA) as a reference. Assays were performed in a 96-well microtiter plate with bonded GF/C filter membrane (Packard UniFilter-96, GF/C) and consisted of 200 μL of homogenate (0.48cmg protein), 25 μL of [^{3}H]imidacloprid (576cpM) and 25 μL of competing ligand. Ligand concentrations used ranged from 0.001 to 10c000cnM and were tested at least in duplicate per competition assay. The assay was started by the addition of homogenate and incubated for 60cmin at room temperature. Bound [^{3}H]imidacloprid was quantified by filtration into a second 96-well filter plate (conditioned with ice-cold 100cmM potassium phosphate buffer, pH 7.4 (including BSA 5cg liter−1)) using a commercial cell harvester (Brandel). After three washing steps (1cml each) with buffer the 96-well filter plates were dried overnight. Each well was then loaded with 25 μL of scintillation cocktail (Microszint-O-Filtercount, Packard) and the plate counted in a Topcount scintillation counter (Packard). Non-specific binding was determined using a final concentration of 10cμM unlabelled imidacloprid. All binding experiments were repeated twice using three replicates per tested ligand concentration. Data were analyzed using a 4 parameter logistic non-linear fitting routine (GraphPad Prism version 7 (GraphPad Software, CA, USA)) in order to calculate I_{50}-values (concentration of unlabelled ligand displacing 50% of [^{3}H]imidacloprid from its binding site).

Functional expression of bee P450s

All bee P450 (see Table S3 for accession numbers) and house fly NADPH-dependent cytochrome P450 reductase (CPR) (GenBank accession number Q07994) genes were obtained by gene synthesis (Geneart, CA, USA) and inserted into the pDEST8 expression vector (Invitrogen). Codon optimization of all bee genes was used to optimize expression in lepidopteran cell lines. The PFastbac1 vector with no inserted DNA was used to produce a control virus. The recombinant baculovirus DNA was constructed and transfected into *Trichoplusia ni* (High five cells, Thermo Fisher) using the Bac-to-Bac baculovirus expression system (Invitrogen) according to the manufacturer's instructions. The titer of the recombinant virus was determined following protocols of the supplier. High Five cells grown to a density of 2×10^6 cells ml^{-1} were co-infected with recombinant baculoviruses containing each bee P450 and CPR with a range of MOI (multiplicity of infection) ratios to identify the optimal conditions. Control cells were co-infected with the baculovirus containing vector with no insert (ctrl-virus) and the recombinant baculovirus expressing CPR using the same MOI ratios. Ferric citrate and δ-aminolevulinic acid hydrochloride were added to a final concentration of 0.1 mM at the time of infection and 24 h after infection to compensate the low levels of endogenous heme in the insect cells. After 48 h, cells were harvested, washed with PBS, and microsomes of the membrane fraction prepared according to standard procedures and stored at −80°C [31]. Briefly, pellets were homogenized for 30 s in 0.1M Na/K-phosphate buffer, pH 7.4 containing 1mM EDTA and DTT and 200mM sucrose using a Fastprep (MP Biomedicals), filtered through miracloth and centrifuging for 10 min at 680 g at 4°C. The supernatant was then centrifuged for 1 h at 100,000 g at 4°C, with the pellet subsequently resuspended in 0.1M Na/K-phosphate buffer, pH 7.6 containing 1mM EDTA and DTT and 10% glycerol using a Dounce tissue grinder. P450 expression and functionality was estimated by measuring CO-difference spectra in reduced samples using a Specord 200 Plus Spectrophotometer (Analytik Jena) and scanning from 500 nm to 400 nm [31]. The protein content of samples was determined using Bradford reagent (Sigma) and bovine serum albumin (BSA) as a reference.

Metabolism assays and UPLC-MS/MS analysis

Metabolism of thiacloprid, imidacloprid and acetamiprid were assayed by incubating each recombinant bee P450/CPR (50-80μg of protein/assay) or ctrl-virus/CPR microsomes in 0.1 M potassium phosphate buffer with an NADPH-regenerating system (Promega; 1.3 mM $NADP^+$, 3.3 mM glucose-6-phosphate, 3.3 mM $MgCl_2$, 0.4 U mL^{-1} glucose-6- phosphate dehydrogenase) and substrate (10-25 μM; 0.78125 – 200 μM for enzyme kinetics) at 30°C for 2 h (*A. mellifera* P450s all insecticides), or 27°C for 45 min (*B. terrestris* P450s for imidacloprid and thiacloprid) or 60 min (*B. terrestris* P450s for acetamiprid). The total assay volume was

200 μL using three replicates for each data point. Microsomes incubated without NADPH served as a control. The assay was stopped by the addition of ice-cold acetonitrile (to 80% final concentration), centrifuged for 10 min at 3000 g and the supernatant subsequently analyzed by tandem mass spectrometry as described previously [18]. For the chromatography on a Waters Acquity HSS T3 column (2.1x50mm, 1.8μm), acetonitrile/water/0.1% formic acid was used as the eluent in gradient mode. For detection and quantification in positive ion mode, the MRM transitions 253 > 186, 269 > 202 (thiacloprid, OH-thiacloprid), 256 > 175, 272 > 191 (imidacloprid, OH-imidacloprid) and 223 > 126, 209 > 126 (acetamiprid and N-desmethyl acetamiprid) were monitored. The peak integrals were calibrated externally against a standard calibration curve. The linear range for the quantification of neonicotinoid insecticides and their hydroxylated (thiacloprid and imidacloprid) and N-desmethylated (acetamiprid) metabolites was 0.1 to 1000 ng mL^{-1}. Recovery rates of parent compounds using microsomal fractions without NADPH were normally close to 100%. Substrate turnover from two independent reactions were plotted versus controls and Michaelis-Menten kinetics determined using GraphPad Prism version 7 (GraphPad Software, CA, USA).

Functional activity of recombinant P450s against fluorescent model substrates

The activity of individual *A. mellifera* and *B. terrestris* recombinant P450s were tested against seven fluorescent model substrates (all purchased from Sigma); 7-methoxy-coumarin (MC), 7-Methoxy-4-(tri-fluoromethyl)-coumarin (MFC), 7-ethoxy-coumarin (EC), 7-benzyloxy-4-(trifluoromethyl)-coumarin (BFC), 7-ethoxy-4-trifluoro-methylcoumarin (EFC), 7-benzyloxymethoxy-4-trifluoromethyl coumarin (BOMFC), and 7-p-methoxy-benzyloxy-4-trifluoro coumarin (MOBFC). Assays were carried out in black flat-bottomed 96-well plates in a 100 μL reaction containing 2 pmol of P450 per 50 μg of protein (*B. terrestris*) or 50 μg/well (*A. mellifera*), 1 mM of NADPH (Sigma) and 50 mM of a model substrate (Sigma). Three replicates were carried out for each data point. P450s incubated without NADPH and wells containing only potassium phosphate buffer served as controls. Samples were incubated at 25°C for 45 min (*B. terrestris*) or 30°C for 30 min (*A. mellifera*). Data were recorded using a SpectraMax Gemini XPS (*B. terrestris*) or a SpectraMax M2 (*A. mellifera*) at the excitation/emission wavelength suitable for each model substrate (MC, EC at 390-465, BFC, MFC at 410-535, EFC at 410-510 and BOMFC and MOBFC at 405-510 nm). As these substrates have a similar emission wavelength to NADPH (460 nm) the reaction was terminated prior to measurement by the addition of 100μL of a stop solution (25% DMSO, 0.05 M Tris/HCL pH10, 5 mM glutathione oxidised, and 0.2 U glutathione reductase). The reactions were incubated at 25°C (*B. terrestris*) or 30°C (*A. mellifera*) for a further 15 min and the data were recorded at the required excitation/emission wavelengths stated above. 7-hydroxy-4-(trifluoromethyl)-coumarin (HFC) (Sigma) was used to generate a standard curve for model substrates BFC, EFC, MFC, MOBFC, and BOMFC and 7-hydroxycoumarin (HC) (Sigma) for model substrates EC and MC. Each compound was diluted to a range of concentrations (0, 5, 10, 15, 20, 30, 50, 60, 80 and 100 pmol) using potassium phosphate buffer. 100 μL of each concentration was added to each well with four replicates for each data point. 100 μL of stop solution was then added and the contents mixed. The florescence was measured as above at the corresponding wavelengths for each model substrate. Microsoft Excel was used to calculate the y intercept for each compound. This was then subtracted from the average fluorescence measurement of each P450 along with the average control measurements.

Transgenic expression of bee P450s in *D. melanogaster*

A. mellifera (*AmCYP9Q1–3*) and *B. terrestris* (*BtCYP9Q4–5*) genes were codon optimized for *D. melanogaster* expression and cloned into the *pUASTattB* plasmid (GenBank: EF362409.1). *pUASTattB-CYP9Q1–3* and *pUASTattB-CYP9Q4–5* constructs were injected into preblastodermal embryos of a *D. melanogaster* strain carrying an *attP* docking site on chromosome 2 (*attP40*) and the *phiC31* integrase gene under the control of the vasa regulatory region on the X chromosome [*y w M(eGFP, vas-int, dmRFP)ZH-2A; P{CaryP} attP40*] [32]. The presence of the transgene was confirmed by PCR and sequencing. Genomic DNA was extracted from pools of 10 flies for each line using the Plant DNeasy Mini kit (QIAGEN) following the manufacturers protocol. 20 ng of this DNA was used as template in PCR using Phusion DNA polymerase (Thermo) following the manufacturers protocol and the primers listed in Table S2. Thermocycling conditions consisted of an initial denaturation step at 98°C for 30 s, followed by 35 cycles of 98°C for 10 s, 55°C for 20 s, 72°C for 1 min, and a final extension at 72°C for 5 min. Products were direct Sanger sequenced using the primers detailed in Table S2. Fly lines were made homozygous for the transgene integrations. The GAL4/UAS system was used to control the expression of bee CYP9Q genes in transgenic *D. melanogaster*. The strain *Act5C-GAL4* [y^1 *w*; P{Act5C-GAL4-w}E1/CyO*] was used to drive the expression of *CYP9Q1–3* and *CYP9Q4–5* genes ubiquitously under the control of the Actin5C regulatory sequence. The *Malp-GAL4* strain [*w[*]; P{w[+mW.hs] = GawB}c42*], which expresses *GAL4* in the Malpighian tubules and specific neuronal cells (ellipsoid body, pars intercerebralis, fan shaped and large field neurons), was used to drive the expression of *CYP9Q3* in these tissues. The *UAS-GFP* strain [w^{1118}*; P*{w^{+mC} *= UAS-GFP.nls}14*] was used to visualize the sites of expression driven by *Act5C-GAL4* and *Malp-GAL4* drivers. Transgene expression was confirmed by qPCR as previously described [33]. Total RNA was extracted from 4 pools of 10 adult flies of each line using the ISOLATE II RNA Mini Kit (Bioline) and reverse transcribed to cDNA using Superscript III reverse transcriptase (Invitrogen) following manufacturer protocols in both cases. PCR reactions (20 μL) contained 10 ng of cDNA, 10 μL of SYBR Green JumpStart Taq Readymix (Sigma), and 0.25 μm of each primer. Samples were run on a Rotor-Gene 6000 (Corbett Research) using temperature cycling conditions of: 2 min at 95°C followed by 40 cycles of 95°C for 15 s, 57°C for 15 s and 72°C for 20 s. Data were analyzed in Microsoft Excel according to the $\Delta\Delta C_T$ method [34] using the *RPL11* reference gene for normalization [33]. Full dose response bioassays were performed by feeding adult female flies a range of insecticide concentrations dissolved in sugar/agar. At least three replicates of 20 flies were carried out for each concentration. LC_{50} values were calculated as above.

Expression profiling of bee P450s

Bees were dissected and total RNA was prepared from tissues of single female bees using the PicoPure RNA Isolation Kit (ThermoFisher) as described by the manufacturer. 0.5 μg were used for cDNA synthesis using iScript (Biorad) according to the manufacturer's instructions. PCR reactions (10 μL) contained 2.5 μL of cDNA (7.8 ng), 5 μL of SsoAdvanced Universal SYBR Green Supermix (BioRad), and 0.25 μM of each primer (Table S2). Samples were run on a CFX384 Real Time System (BioRad) using the temperature cycling conditions of: 3 min at 95°C followed by 39 cycles of 95°C for 15 s, 64°C for 15 s and 60°C for 15 s. A final melt-curve step was included post-PCR (ramping from 65-95°C by 0.5°C every 5 s) to confirm the absence of any non-specific amplification. The efficiency of PCR for each primer pair was assessed using a serial dilution of 25 ng to 0.04 ng of cDNA. Each qPCR experiment consisted of at least 7 independent biological replicates with three technical replicates for each. Data were analyzed according to the $\Delta\Delta C_T$ method [34] using qbase+ Version: 3.1 (Biogazelle). The expression level was normalized to two validated reference genes [35–37] for each species. *Rpl32* (ribosomal protein L32), *GADPH* (glyceraldehyde 3-phosphate dehydrogenase), PAL2 (phospholipase A2) and *EEF1A* (elongation factor 1-alpha) of the honeybee and bumble bee respectively (Table S2). *In situ* hybridization with antibody labeled RNA probes was used to visualize the expression of *CYP9Q3* in the brain and Malpighian tubules of honeybees. Fragments of ~700 bp were amplified from honeybee cDNA by PCR using gene-specific primers (Table S2) containing the T7 promoter sequence at the end and served as templates for synthesis using the T7 RNA polymerase and digoxigenin-labeled ribonucleotides. Digoxigenin-labeled riboprobes were purified and hydrolyzed into 100-400 bp fragments with 0.1 M sodium carbonate. Tissues from cold-anaesthetized bees were then dissected in PBS, fixed overnight in 4% paraformaldehyde and dehydrated in a methanol series. Before hybridization tissues were rehydrated in PBS/0.1% Tween, pre-incubated overnight at 55°C in hybridization buffer (50% formamide, 5xSSC, 0.1% Tween, 100 μg ml^{-1} yeast tRNA, 200 μg ml^{-1} salmon sperm, 50 μg ml^{-1} heparin) and then hybridized with the diluted riboprobes (1.0-4.0 ug ml^{-1} in hybridization buffer) at 55°C. After extensive post-hybridization stringency washes samples were pre-blocked in 1% BSA for at least 1 h prior to overnight incubation with the pre-adsorbed alkaline phosphatase labeled antidigoxygenin antibody (1:2000 dilution in PBS/1% BSA/0.1% Tween). The signal was visualized with NBT/BCIP alkaline phosphatase substrates according to the manufacturer's instructions.

QUANTIFICATION AND STATISTICAL ANALYSIS

All statistical analyses were performed in GraphPad Prism 7 (GraphPad Software) apart from qPCR analsyses, which were performed in qbase+ Version 3.1 (Biogazelle). Significant differences in expression in all qPCR experiments were determined using one-way ANOVA with post hoc testing (Benjamini and Hochberg). Significant differences in activity of recombinant P450s against thiacloprid and imidacloprid was determined using a Welch's t test. Statistical details of experiments (value of n, precision measures and definitions of significance) are provided in figure legends.

DATA AND SOFTWARE AVAILABILITY

The sequences reported in this paper are all available in online sequence repositories (see Table S3).

Current Biology, Volume *28*

Supplemental Information

Unravelling the Molecular Determinants

of Bee Sensitivity to Neonicotinoid Insecticides

Cristina Manjon, Bartlomiej J. Troczka, Marion Zaworra, Katherine Beadle, Emma Randall, Gillian Hertlein, Kumar Saurabh Singh, Christoph T. Zimmer, Rafael A. Homem, Bettina Lueke, Rebecca Reid, Laura Kor, Maxie Kohler, Jürgen Benting, Martin S. Williamson, T.G. Emyr Davies, Linda M. Field, Chris Bass, and Ralf Nauen

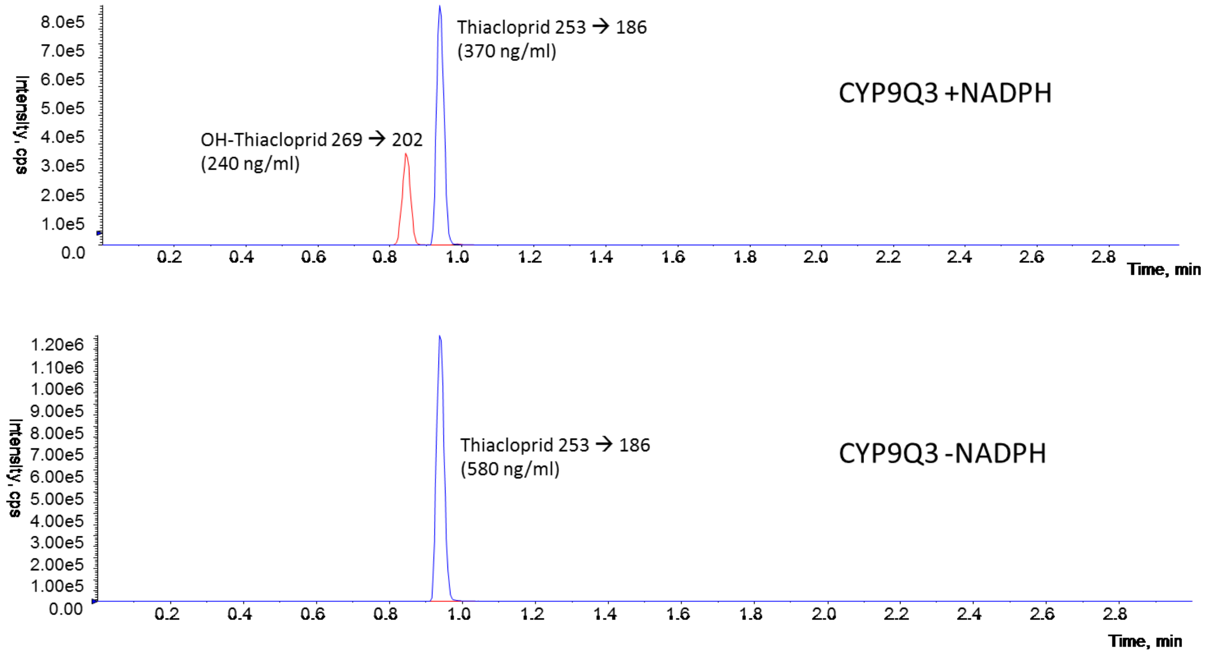

Figure S1. LC-MS/MS analysis of thiacloprid metabolism by CYP9Q3. Related to Figure 2. LC-MS analysis of thiacloprid metabolism. Typical MRM chromatograms of the CYP9Q3 catalysed formation of OH-thiacloprid with and without NADPH. Ion transition of thiacloprid $[M+H]^+$ 253 and OH-thiacloprid $[M+H]^+$ 269 to their fragments m/z 186 and m/z 202 are measured, respectively.

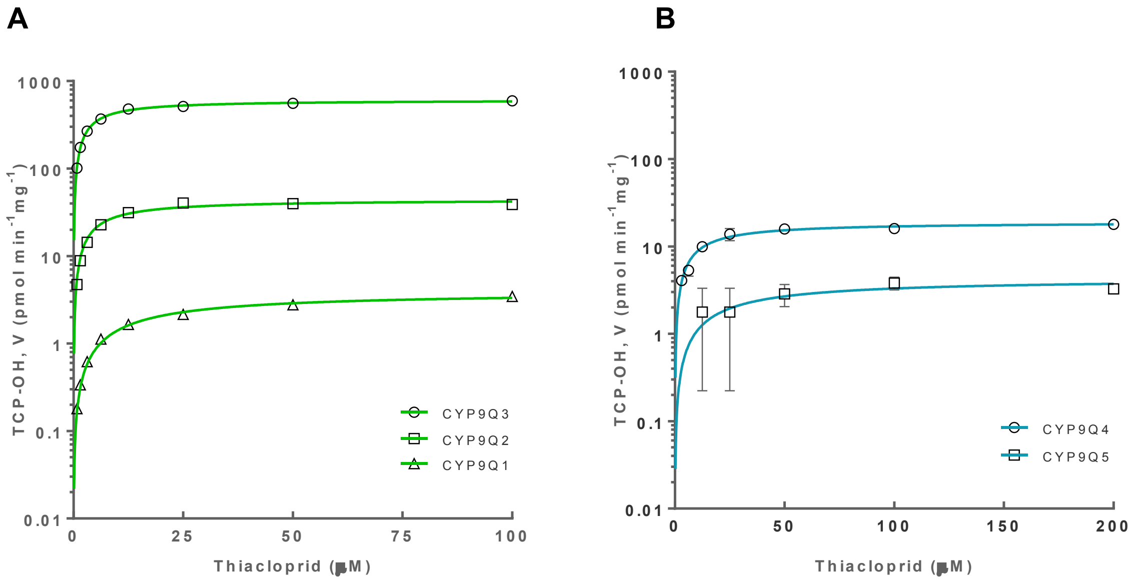

	CYP9Q1	CYP9Q2	CYP9Q3
V_{max}	3,92 ±0,08	44,31±1,01	607,8±5,48
K_m	17,83±1,08	5,65±0,49	3,92±0,14

	CYP9Q4	CYP9Q5
V_{max}	18,94±0,64	4,29±0,71
K_m	11,82±1,51	30,04±14,85

Figure S2. Michaelis-Menten kinetics of thiacloprid hydroxylation by *A. mellifera* and *B. terrestris* metabolising P450s analysed by non-linear regression. Related to Figure 2. A, B, Michaelis-Menten kinetics plots of thiacloprid hydroxylation catalyzed by AmCYP9Q1-3 **(A)** and BtCYP9Q4-5 **(B)**. The apparent K_m and V_{max} values for thiacloprid are indicated below the respective graphs. Data points are mean values ± SD (n=3).

	Bt CYP9P1	Bt CYP9P2	Bt CYP9R1	Bt CYP9Q4	Bt CYP9Q5	Am CYP9Q1	Am CYP9Q3	Am CYP9Q2
Bt CYP9P1		63.953%	41.454%	37.795%	37.795%	37.305%	36.471%	38.235%
Bt CYP9P2	63.953%		41.765%	40.354%	40.748%	37.305%	37.451%	39.412%
Bt CYP9R1	41.454%	41.765%		42.185%	42.938%	38.716%	41.892%	42.402%
Bt CYP9Q4	37.795%	40.354%	42.185%		93.143%	55.340%	56.840%	61.163%
Bt CYP9Q5	37.795%	40.748%	42.938%	93.143%		54.563%	57.225%	61.726%
Am CYP9Q1	37.305%	37.305%	38.716%	55.340%	54.563%		53.488%	56.589%
Am CYP9Q3	36.471%	37.451%	41.892%	56.840%	57.225%	53.488%		57.500%
Am CYP9Q2	38.235%	39.412%	42.402%	61.163%	61.726%	56.589%	57.500%	

Figure S3. Heat map showing the levels of sequence identity between *A. mellifera CYP9Q1-3* and *B. terrestris CYP9* genes. Related to Figure 2.

Application	Insecticide	Synergist	LD_{50} (µg/bee)	95% CI	Slope	± SE	Synergism Ratio
Topical	Imidacloprid	None	0.38	0.12 - 1.45	0.6	0.11	n/a
	Thiacloprid	None	>100	n/a	n/a	n/a	n/a
Oral	Imidacloprid	None	0.038	0.012 - 0.075	1.5	0.44	n/a
		PBO	0.032	0.016 - 0.05	1.9	0.41	1.2
	Thiacloprid	None	19.68	13.45 - 26.88	1.8	0.26	n/a
		PBO	4.73	2.55 - 7.71	1.4	0.24	4.2

Table S1. Sensitivity of *Bombus terrestris* to imidacloprid and thiacloprid in insecticide bioassays. Related to Figure 1. Neonicotinoid acute contact and acute oral LD_{50} values (±95% confidence intervals) and slope (±SE) for *Bombus terrestris* 48 hours after application of insecticide. Synergism ratio is also shown, where the P450 inhibitor piperonyl butoxide (PBO) was used.

Primers	**Sequence**	**Use**
**AmCyp9Q3 F1**	5'-GATGTGCGTCGAGAGTTTCC-3'	qPCR (CYP9Q3)
**AmCyp9Q3 R1**	5'-CTGTCCGGGTCGAATTTGTC-3'	qPCR (CYP9Q3)
**AmCyp9Q2 F1**	5'-ATGGAAGGAGCACAGGAACA-3'	qPCR (CYP9Q2)
**AmCyp9Q2 R1**	5'-ACGTCGTTGGTGTATCTGGT-3'	qPCR (CYP9Q2)
**AmCyp9Q1 F1**	5'-GGAGGAGGGGAAGAGAGGTA -3'	qPCR (CYP9Q1)
**AmCyp9Q1 R1**	5'-CCTCCTGAAGCCTCTGTTGA-3'	qPCR (CYP9Q1)
**AmRpl32 F1**	5'-AGTAAATTAAAGAGAAACTGGCGTAA-3'	qPCR (reference gene)
**AmRpl32 R1**	5'-TAAAACTTCCAGTTCCTTGACATTAT-3'	qPCR (reference gene)
**AmGADPH F1**	5'-ACCTTCTGCAAAATTATGGCGA-3'	qPCR (reference gene)
**AmGADPH R1**	5'-CACCTTTGCCAAGTCTAACTGTTAAG-3'	qPCR (reference gene)
**BtCyp9Q4 F1**	5’-TATTCCACCAACGCCACTGT-3’	qPCR (CYP9Q4)
**BtCyp9Q4 R1**	5’-GGTCCACTTCCTTGTATGCG-3’	qPCR (CYP9Q4)
**BtCyp9Q5 F1**	5’-CCTACGATGCTCTAAGCGAGATG-3’	qPCR (CYP9Q5)
**BtCyp9Q5 R1**	5’-ATTCTCGTAATATTGAGGATCGCG-3’	qPCR (CYP9Q5)
**BtPal F1**	5'-TGTCGGTATCTACGCGCCTG-3'	qPCR (reference gene)
**BtPal R1**	5'-TTGGTGGATGCTTGTCAGTC-3'	qPCR (reference gene)
**BtEEF1A F1**	5'-AGAATGGACAAACCCGTGAG-3'	qPCR (reference gene)
**BtEEF1A R1**	5'-CACAAATGCTACCGCAACAG-3'	qPCR (reference gene)
**D099 pUAST F**	TCACTGGAACTAGGCTAGCA-3'	Sequence validation of transgenic flies
**D102 pUAST F**	5'-GGATCCAAGCTTGCATGCCTG-3'	sequence validation of transgenic flies
**D100 pUAST R**	5'-AAAGGCATTCCACCACTGCT-3'	sequence validation of transgenic flies
**D101 pUAST R**	5'-CCACCACTGCTCCCATTCAT-3'	sequence validation of transgenic flies
**AmCyp9Q3 F3**	5'-TGGAAGGAGCACAGGAACAT-3'	in situ hybridisation (CYP9Q3)
**AmCyp9Q3 R6-T7**	5'-TAATACGACTCACTATAGGGAGATGATCACGGCGTCCATGTAT-3'	In situ hybridisation (CYP9Q3)

Table S2. Sequence of oligonucleotide primers for, PCR, qRT-PCR and *in situ* hybridization used in this study. Related to STAR methods.

Species	Gene name	Accession Number
Bombus terrestris	CYP9Q4	XP_003393377
Bombus terrestris	CYP9Q5	XP_003393376.1
Bombus terrestris	CYP9P1	XP_020718545.1
Bombus terrestris	CYP9P2	XP_003393388.3
Bombus terrestris	CYP9R1	XP_003393379.1
Apis mellifera	CYP9Q1	XP_006562364
Apis mellifera	CYP9Q2	XP_392000
Apis mellifera	CYP9Q3	XP_006562363
Apis mellifera	CYP9R1	GB16803
Apis mellifera	CYP9S1	XP_016771487
Apis mellifera	CYP336A1	XP_001119981
Apis mellifera	CYP9P1	XP_006562365
Apis mellifera	CYP9P2	GB19055
Apis mellifera	CYP6AQ1	NP_001191991
Apis mellifera	CYP6AR1	XP_623362
Apis mellifera	CYP6AS1	GB16899
Apis mellifera	CYP6AS2	GB19197
Apis mellifera	CYP6AS3	GB15681
Apis mellifera	CYP6AS4	XP_395671
Apis mellifera	CYP6AS5	DQ232888
Apis mellifera	CYP6AS7	XP_006565064
Apis mellifera	CYP6AS8	XP_006565076
Apis mellifera	CYP6AS10	XP_016771320
Apis mellifera	CYP6AS11	XP_016771191
Apis mellifera	CYP6AS12	XP_397347
Apis mellifera	CYP6AS13	GB17831
Apis mellifera	CYP6AS15	XP_623595
Apis mellifera	CYP6AS17	XP_006565063
Apis mellifera	CYP6AS18	XP_006565063
Apis mellifera	CYP6BC1	XP_016766476
Apis mellifera	CYP6BD1	XP_006564499
Apis mellifera	CYP6BE1	XP_624795

Table S3. Accession numbers of P450 sequences functionally expressed in this study. Related to STAR methods.
